# *cis*-Bis(*O*-methyl­dithio­carbonato-κ^2^ *S*,*S*′)bis­(tri­phenyl­phosphane-κ*P*)ruthenium(II)

**DOI:** 10.1107/S1600536813016735

**Published:** 2013-06-22

**Authors:** Cintya Valerio-Cárdenas, Simón Hernández-Ortega, Reyna Reyes-Martínez, David Morales-Morales

**Affiliations:** a Instituto de Química, Universidad Nacional Autónoma de México, Circuito Exterior, Ciudad Universitaria, México, D.F., 04510, Mexico

## Abstract

In the title compound, [Ru(CH_3_OCS_2_)_2_(C_18_H_15_P)_2_], the Ru^II^ atom is in a distorted octa­hedral coordination by two xanthate anions (CH_3_OCS_2_) and two tri­phenyl­phosphane (PPh_3_) ligands. Both bidentate xanthate ligands coordinate the Ru^II^ atom with two slightly different Ru—S bond lengths but with virtually equal bite angles [71.57 (4) and 71.58 (3)°]. The packing of the complexes is assured by C—H⋯O and C—H⋯π inter­actions.

## Related literature

For complexes with metal-S and metal-P bonds, see: Lu *et al.* (2003 ▶); Wang *et al.* (2010 ▶). For ruthenium complexes with di­thiol­ate ligands, see: Bag *et al.* (1990 ▶); Liu *et al.* (2005 ▶); Noda *et al.* (2006 ▶); Wu *et al.* (2009 ▶).
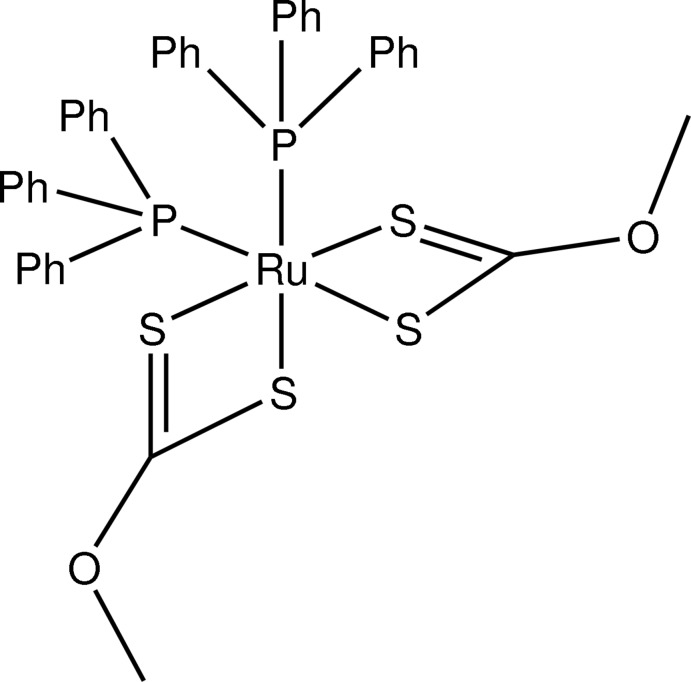


## Experimental

### 

#### Crystal data

[Ru(C_2_H_3_OS_2_)_2_(C_18_H_15_P)_2_]
*M* *_r_* = 839.94
Orthorhombic, 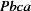

*a* = 10.7285 (3) Å
*b* = 18.5470 (4) Å
*c* = 38.0785 (9) Å
*V* = 7576.9 (3) Å^3^
*Z* = 8
Mo *K*α radiation
μ = 0.75 mm^−1^
*T* = 298 K
0.32 × 0.21 × 0.18 mm

#### Data collection

Bruker SMART APEX CCD area-detector diffractometer
Absorption correction: multi-scan (*SADABS*; Bruker, 2007 ▶) *T*_min_ = 0.665, *T*_max_ = 0.745
31337 measured reflections
6924 independent reflections
4970 reflections with *I* > 2σ(*I*)
*R*_int_ = 0.048

#### Refinement

*R*[*F* ^2^ > 2σ(*F* ^2^)] = 0.039
*wR*(*F* ^2^) = 0.097
*S* = 1.03
6924 reflections
444 parameters
H-atom parameters constrained
Δρ_max_ = 0.45 e Å^−3^
Δρ_min_ = −0.52 e Å^−3^

### 

Data collection: *APEX2* (Bruker, 2007 ▶); cell refinement: *SAINT* (Bruker, 2007 ▶); data reduction: *SAINT*; program(s) used to solve structure: *SHELXS97* (Sheldrick, 2008 ▶); program(s) used to refine structure: *SHELXL97* (Sheldrick, 2008 ▶); molecular graphics: *ORTEP-3 for Windows* (Farrugia, 2012 ▶) and *DIAMOND* (Brandenburg, 2006 ▶); software used to prepare material for publication: *SHELXTL* (Sheldrick, 2008 ▶) and *PLATON* (Spek, 2009 ▶).

## Supplementary Material

Crystal structure: contains datablock(s) I, global. DOI: 10.1107/S1600536813016735/vn2074sup1.cif

Structure factors: contains datablock(s) I. DOI: 10.1107/S1600536813016735/vn2074Isup2.hkl

Additional supplementary materials:  crystallographic information; 3D view; checkCIF report

## Figures and Tables

**Table 1:** Selected bond lengths (Å)

Ru1—P1	2.3180 (9)
Ru1—P2	2.3493 (9)
Ru1—S1	2.4015 (10)
Ru1—S2	2.4530 (10)
Ru1—S3	2.3981 (9)
Ru1—S4	2.4426 (9)

**Table 2:** Hydrogen-bond geometry (Å, °) *Cg*1 is the centroid of the C25–C30 ring.

*D*—H⋯*A*	*D*—H	H⋯*A*	*D*⋯*A*	*D*—H⋯*A*
C11—H11⋯O5^i^	0.93	2.51	3.387 (5)	157
C40—H40⋯*Cg* ^ii^	0.93	2.85	3.521 (4)	130

Symmetry codes: (i) 
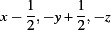
; (ii) 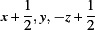.

**Fig. 1.**
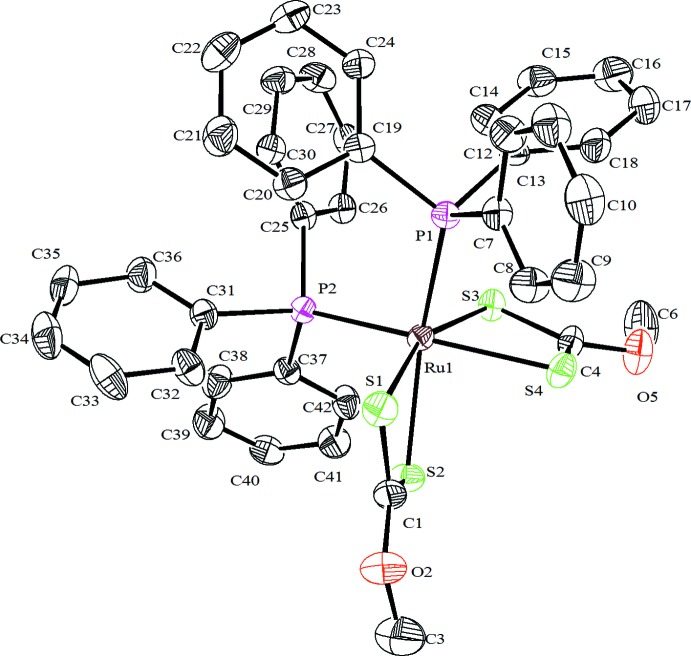
Molecular structure of the title compound, showing 30% probability displacement ellipsoids. The hydrogen atoms have been omitted for clarity

**Fig. 2.**
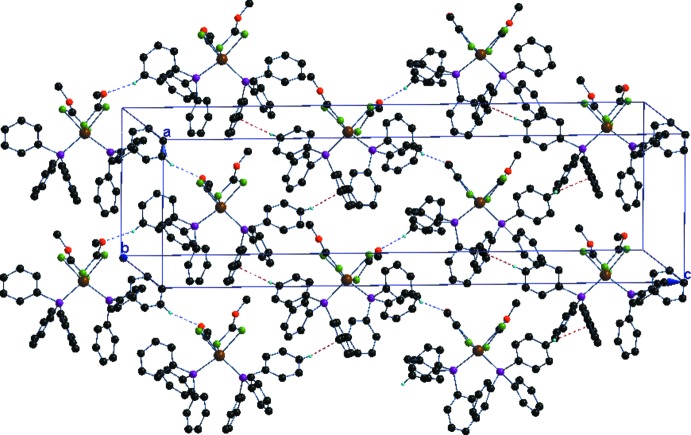
Layer arrangement generated by C—H···O and C—H···π interactions. Hydrogen bond interactions are shown by dashed lines.

## supplementary crystallographic information

### Comment

Complexes containing metal-S and metal-P bonds are of great interest due to their potential role in homogeneous catalysis (Lu *et al.*, 2003; Wang *et al.*, 2010). In this context, complexes with Ru—S bonds may serve in hydrotreating processes of different fractions of oil and as functional models for Fe—S proteins. Some common sulfur-ligands used to coordinate Ru^II^ are dithiolates as dithiocarbamates (*R*_2_NCS_2_^-^), xanthates (*R*OCS_2_^-^) and dithiophosphates ((*R*O)_2_PS_2_^-^). Examples of Ru(II) complexes with dithiolates reported previously include *trans*-[Ru(PPh_3_)_2_(S_2_COPr)_2_], *cis*-[Ru(PPh_3_)_2_(S_2_COPr)_2_], *cis*-[Ru(PPh_3_)_2_(S_2_CO*^i^*Pr)_2_] (Wu *et al.*, 2009; Bag *et al.*, 1990), *cis*-[Ru(PPh_3_)_2_(S_2_COEt)_2_] (Noda *et al*., 2006) and *cis*-[Ru(PPh_3_)_2_(S_2_P(OEt)_2_)_2_] (Liu *et al.*, 2005).

We report here the crystal structure of *cis*-bis(*O*-Methyldithiocarbonato)-bis(triphenylphosphane)ruthenium (II) *cis*-[Ru(PPh_3_)_2_(S_2_COMe)_2_] of which the molecular structure is shown in Fig. 1.

The title complex is mononuclear and the ruthenium center is found in a distorted octahedral geometry. The coordination sphere is composed of two triphenylphosphane ligands (PPh_3_) and two xanthate ligands (S_2_COMe) arranged in a *cis* conformation. The two xanthate ligands coordinate the Ru^II^ atom in a bidentated manner with Ru—S distances of 2.4015 (10) and 2.4528 (11) Å for one ligand, and 2.3982 (10) and 2.4425 (11) Å for the other. Such slightly different Ru-S distances for the bidentate xanthate ligand are also found in the analogue compounds *cis*-[Ru(PPh_3_)_2_(S_2_CO*^i^*Pr)_2_] (Wu *et al.*, 2009) and *cis*-[Ru(PPh_3_)_2_(S_2_COEt)_2_] (Noda *et al.*, 2006). The two bite angles of the chelating xanthate ligands are nearly the same: 71.57 (4)° for S1—Ru1—S2 and 71.58 (4)° for S3—Ru1—S4. The two PPh_3_ ligands are arranged in a *cis* conformation with a P1—Ru1—P2 angle of 100.95 (3)°. The Ru—P distances are 2.3180 (8) Å for Ru1—P1 and 2.3494 (8) Å for Ru1—P2, respectively. These distances are similar to those found in related compounds. There are weak non-covalent interactions [C11—H11···O5 and C40—H40···π], which produce a layer arrangement parallel to the *ac* plane (Fig. 2).

### Experimental

A mixture of carbon disulfide CS_2_ (0.06 ml) and sodium hydroxide KOH (0.003 g, 0.052 mmol) in methanol (30 ml) was stirred a room temperature overnight. Then [RuHCl(CO)(PPh_3_)_3_] (0.050 g, 0.052 mmol) was added and the yellow solution was set to reflux for 3 h. Brown crystals suitable for single-crystal X-ray diffraction analysis were obtained by slow evaporation of the solvent from a saturated solution of the title compound.^1^H RMN (300 MHz, CDCl_3_) δ: 1.18 (s, 6H, –CH_3_), 7.0–7.6 (m, PPh_3_). ^31^P {^1^H} NMR (121 MHz, CDCl_3_) δ: 43.33 (*s*).

### Refinement

H atoms were included in calculated position (C—H = 0.93 Å for aromatic H, and C—H = 0.96 Å for methyl H), and refined using a riding model with *U*_iso_(H) = 1.2 *U*_eq_ of the carrier atoms. 5 badly fitting reflections were omitted from the final refinement.

### Crystal data

**Table:**

[Ru(C_2_H_3_OS_2_)_2_(C_18_H_15_P)_2_]	*D*_x_ = 1.473 Mg m^−^^3^
*M**_r_* = 839.94	Mo *K*α radiation, λ = 0.71073 Å
Orthorhombic, *P**b**c**a*	Cell parameters from 4576 reflections
*a* = 10.7285 (3) Å	θ = 2.3–22.7°
*b* = 18.5470 (4) Å	µ = 0.75 mm^−^^1^
*c* = 38.0785 (9) Å	*T* = 298 K
*V* = 7576.9 (3) Å^3^	Prism, brown
*Z* = 8	0.32 × 0.21 × 0.18 mm
*F*(000) = 3440	

### Data collection

**Table:**

Bruker SMART APEX CCD area-detector diffractometer	4970 reflections with *I* > 2σ(*I*)
Detector resolution: 0.83 pixels mm^-1^	*R*_int_ = 0.048
ω scans	θ_max_ = 25.4°, θ_min_ = 2.1°
Absorption correction: multi-scan (*SADABS*; Bruker, 2007)	*h* = −12→12
*T*_min_ = 0.665, *T*_max_ = 0.745	*k* = −22→11
31337 measured reflections	*l* = −45→44
6924 independent reflections	

### Refinement

**Table:**

Refinement on *F*^2^	0 restraints
Least-squares matrix: full	Hydrogen site location: inferred from neighbouring sites
*R*[*F*^2^ > 2σ(*F*^2^)] = 0.039	H-atom parameters constrained
*wR*(*F*^2^) = 0.097	*w* = 1/[σ^2^(*F*_o_^2^) + (0.0395*P*)^2^ + 3.2859*P*] where *P* = (*F*_o_^2^ + 2*F*_c_^2^)/3
*S* = 1.03	(Δ/σ)_max_ = 0.001
6924 reflections	Δρ_max_ = 0.45 e Å^−^^3^
444 parameters	Δρ_min_ = −0.52 e Å^−^^3^

### Special details

**Table:**

Geometry. All e.s.d.'s (except the e.s.d. in the dihedral angle between two l.s. planes) are estimated using the full covariance matrix. The cell e.s.d.'s are taken into account individually in the estimation of e.s.d.'s in distances, angles and torsion angles; correlations between e.s.d.'s in cell parameters are only used when they are defined by crystal symmetry. An approximate (isotropic) treatment of cell e.s.d.'s is used for estimating e.s.d.'s involving l.s. planes.

### Fractional atomic coordinates and isotropic or equivalent isotropic displacement parameters (Å^2^)

**Table:**

	*x*	*y*	*z*	*U*_iso_*/*U*_eq_	
Ru1	0.49018 (2)	0.22296 (2)	0.12872 (2)	0.03379 (10)	
S1	0.53986 (10)	0.34881 (5)	0.13361 (3)	0.0530 (3)	
S2	0.65542 (9)	0.23693 (6)	0.17272 (3)	0.0584 (3)	
S3	0.51741 (8)	0.09579 (5)	0.12028 (2)	0.0433 (2)	
S4	0.66125 (9)	0.20447 (5)	0.08736 (3)	0.0510 (3)	
P1	0.36233 (8)	0.23769 (5)	0.08017 (2)	0.0360 (2)	
P2	0.34169 (8)	0.20338 (5)	0.17305 (2)	0.0349 (2)	
C1	0.6547 (3)	0.3238 (2)	0.16107 (10)	0.0559 (11)	
O2	0.7348 (3)	0.3757 (2)	0.17080 (9)	0.0946 (11)	
C3	0.8371 (5)	0.3540 (3)	0.19023 (15)	0.123 (2)	
H3A	0.8095	0.3302	0.2112	0.185*	
H3B	0.8861	0.3954	0.1964	0.185*	
H3C	0.8868	0.3214	0.1765	0.185*	
C4	0.6404 (3)	0.1166 (2)	0.09494 (9)	0.0465 (9)	
O5	0.7202 (3)	0.06898 (15)	0.08116 (8)	0.0700 (8)	
C6	0.6966 (5)	−0.0058 (2)	0.08750 (13)	0.0940 (17)	
H6A	0.6917	−0.0142	0.1123	0.141*	
H6B	0.7630	−0.0341	0.0777	0.141*	
H6C	0.6191	−0.0193	0.0767	0.141*	
C7	0.4101 (3)	0.30357 (18)	0.04616 (9)	0.0411 (8)	
C8	0.5228 (4)	0.3396 (2)	0.04596 (10)	0.0560 (11)	
H8	0.5794	0.3318	0.0641	0.067*	
C9	0.5527 (4)	0.3872 (2)	0.01924 (11)	0.0688 (12)	
H9	0.6290	0.4110	0.0196	0.083*	
C10	0.4715 (5)	0.3995 (2)	−0.00764 (12)	0.0671 (12)	
H10	0.4920	0.4317	−0.0255	0.081*	
C11	0.3592 (4)	0.3641 (2)	−0.00824 (10)	0.0633 (12)	
H11	0.3038	0.3720	−0.0266	0.076*	
C12	0.3284 (4)	0.3168 (2)	0.01837 (9)	0.0531 (10)	
H12	0.2518	0.2933	0.0178	0.064*	
C13	0.3449 (3)	0.15609 (18)	0.05361 (9)	0.0404 (8)	
C14	0.2740 (3)	0.09938 (19)	0.06594 (10)	0.0506 (10)	
H14	0.2295	0.1049	0.0867	0.061*	
C15	0.2675 (4)	0.0341 (2)	0.04797 (12)	0.0623 (11)	
H15	0.2183	−0.0033	0.0565	0.075*	
C16	0.3334 (4)	0.0255 (2)	0.01798 (14)	0.0738 (14)	
H16	0.3297	−0.0181	0.0060	0.089*	
C17	0.4059 (4)	0.0807 (3)	0.00510 (12)	0.0733 (13)	
H17	0.4507	0.0743	−0.0156	0.088*	
C18	0.4124 (4)	0.1461 (2)	0.02284 (10)	0.0555 (10)	
H18	0.4618	0.1831	0.0141	0.067*	
C19	0.2034 (3)	0.27155 (17)	0.08636 (9)	0.0382 (8)	
C20	0.1884 (3)	0.33041 (18)	0.10886 (10)	0.0473 (9)	
H20	0.2575	0.3498	0.1202	0.057*	
C21	0.0722 (4)	0.3602 (2)	0.11449 (11)	0.0633 (11)	
H21	0.0630	0.3994	0.1295	0.076*	
C22	−0.0300 (4)	0.3315 (2)	0.09773 (13)	0.0683 (13)	
H22	−0.1087	0.3507	0.1020	0.082*	
C23	−0.0170 (4)	0.2751 (2)	0.07487 (12)	0.0617 (12)	
H23	−0.0863	0.2569	0.0632	0.074*	
C24	0.0991 (3)	0.2452 (2)	0.06914 (9)	0.0477 (9)	
H24	0.1074	0.2069	0.0535	0.057*	
C25	0.2161 (3)	0.14138 (17)	0.15982 (8)	0.0363 (8)	
C26	0.2377 (3)	0.06684 (18)	0.15944 (9)	0.0432 (9)	
H26	0.3124	0.0487	0.1681	0.052*	
C27	0.1490 (4)	0.0200 (2)	0.14633 (9)	0.0525 (10)	
H27	0.1652	−0.0292	0.1459	0.063*	
C28	0.0367 (4)	0.0455 (2)	0.13385 (10)	0.0571 (11)	
H28	−0.0227	0.0137	0.1252	0.069*	
C29	0.0132 (3)	0.1184 (2)	0.13428 (10)	0.0534 (10)	
H29	−0.0627	0.1358	0.1261	0.064*	
C30	0.1019 (3)	0.16590 (19)	0.14680 (9)	0.0433 (9)	
H30	0.0852	0.2151	0.1466	0.052*	
C31	0.2568 (3)	0.27645 (17)	0.19566 (9)	0.0409 (8)	
C32	0.3135 (4)	0.34300 (18)	0.19887 (9)	0.0524 (10)	
H32	0.3907	0.3511	0.1885	0.063*	
C33	0.2549 (5)	0.3980 (2)	0.21766 (11)	0.0728 (14)	
H33	0.2923	0.4432	0.2190	0.087*	
C34	0.1448 (5)	0.3869 (3)	0.23398 (12)	0.0763 (14)	
H34	0.1070	0.4239	0.2465	0.092*	
C35	0.0896 (4)	0.3207 (3)	0.23186 (12)	0.0719 (13)	
H35	0.0149	0.3124	0.2435	0.086*	
C36	0.1439 (4)	0.2660 (2)	0.21249 (10)	0.0573 (11)	
H36	0.1039	0.2217	0.2107	0.069*	
C37	0.4031 (3)	0.15415 (17)	0.21204 (9)	0.0390 (8)	
C38	0.3472 (4)	0.1590 (2)	0.24458 (10)	0.0571 (11)	
H38	0.2792	0.1895	0.2475	0.068*	
C39	0.3904 (4)	0.1191 (2)	0.27324 (10)	0.0664 (12)	
H39	0.3512	0.1236	0.2949	0.080*	
C40	0.4892 (4)	0.0738 (2)	0.26969 (11)	0.0567 (11)	
H40	0.5172	0.0468	0.2887	0.068*	
C41	0.5467 (4)	0.0685 (2)	0.23786 (11)	0.0586 (11)	
H41	0.6153	0.0383	0.2353	0.070*	
C42	0.5037 (3)	0.1079 (2)	0.20913 (10)	0.0538 (10)	
H42	0.5435	0.1031	0.1876	0.065*	

### Atomic displacement parameters (Å^2^)

**Table:**

	*U*^11^	*U*^22^	*U*^33^	*U*^12^	*U*^13^	*U*^23^
Ru1	0.03219 (16)	0.03129 (16)	0.03787 (17)	−0.00165 (12)	0.00045 (12)	0.00142 (12)
S1	0.0552 (6)	0.0367 (5)	0.0671 (7)	−0.0079 (4)	0.0044 (5)	−0.0017 (5)
S2	0.0481 (6)	0.0714 (7)	0.0557 (6)	−0.0006 (5)	−0.0137 (5)	−0.0007 (5)
S3	0.0465 (5)	0.0367 (5)	0.0467 (5)	0.0009 (4)	0.0086 (4)	0.0022 (4)
S4	0.0430 (5)	0.0498 (6)	0.0601 (6)	−0.0041 (4)	0.0154 (5)	0.0044 (5)
P1	0.0370 (5)	0.0336 (5)	0.0375 (5)	−0.0023 (4)	−0.0007 (4)	0.0014 (4)
P2	0.0361 (5)	0.0301 (5)	0.0384 (5)	−0.0001 (4)	0.0011 (4)	0.0007 (4)
C1	0.046 (2)	0.062 (3)	0.060 (3)	−0.021 (2)	0.006 (2)	−0.016 (2)
O2	0.064 (2)	0.118 (3)	0.102 (3)	−0.024 (2)	−0.013 (2)	−0.027 (2)
C3	0.094 (4)	0.177 (7)	0.099 (5)	−0.028 (4)	−0.015 (4)	−0.028 (4)
C4	0.042 (2)	0.051 (2)	0.046 (2)	0.0094 (18)	0.0093 (17)	0.0016 (18)
O5	0.076 (2)	0.0558 (18)	0.079 (2)	0.0093 (15)	0.0323 (16)	0.0025 (15)
C6	0.128 (5)	0.052 (3)	0.103 (4)	0.019 (3)	0.038 (3)	−0.007 (3)
C7	0.047 (2)	0.0374 (19)	0.039 (2)	0.0040 (17)	0.0030 (17)	0.0055 (16)
C8	0.059 (3)	0.057 (3)	0.051 (2)	−0.012 (2)	−0.003 (2)	0.013 (2)
C9	0.073 (3)	0.068 (3)	0.066 (3)	−0.021 (2)	0.006 (3)	0.019 (2)
C10	0.095 (4)	0.051 (3)	0.055 (3)	0.001 (2)	0.017 (3)	0.018 (2)
C11	0.080 (3)	0.063 (3)	0.047 (2)	0.012 (2)	0.001 (2)	0.015 (2)
C12	0.057 (2)	0.055 (2)	0.048 (2)	0.001 (2)	0.000 (2)	0.0090 (19)
C13	0.040 (2)	0.039 (2)	0.043 (2)	0.0040 (16)	−0.0096 (17)	−0.0039 (16)
C14	0.059 (2)	0.043 (2)	0.050 (2)	−0.0051 (19)	−0.004 (2)	0.0008 (18)
C15	0.062 (3)	0.042 (2)	0.082 (3)	−0.001 (2)	−0.016 (2)	−0.008 (2)
C16	0.071 (3)	0.049 (3)	0.102 (4)	0.009 (2)	−0.015 (3)	−0.028 (3)
C17	0.068 (3)	0.077 (3)	0.074 (3)	0.015 (3)	0.005 (3)	−0.033 (3)
C18	0.050 (2)	0.056 (3)	0.061 (3)	0.0053 (19)	0.005 (2)	−0.011 (2)
C19	0.040 (2)	0.0341 (19)	0.041 (2)	−0.0022 (16)	−0.0011 (16)	0.0051 (16)
C20	0.049 (2)	0.038 (2)	0.054 (2)	−0.0010 (17)	−0.0020 (19)	−0.0007 (18)
C21	0.063 (3)	0.050 (2)	0.077 (3)	0.011 (2)	0.004 (2)	−0.008 (2)
C22	0.049 (3)	0.064 (3)	0.092 (4)	0.015 (2)	0.001 (2)	0.010 (3)
C23	0.040 (2)	0.066 (3)	0.078 (3)	−0.005 (2)	−0.005 (2)	0.004 (2)
C24	0.042 (2)	0.051 (2)	0.051 (2)	−0.0043 (18)	−0.0016 (18)	−0.0041 (19)
C25	0.043 (2)	0.0320 (19)	0.0342 (19)	−0.0030 (15)	0.0059 (16)	0.0031 (15)
C26	0.049 (2)	0.034 (2)	0.046 (2)	−0.0004 (17)	0.0036 (18)	0.0013 (16)
C27	0.072 (3)	0.037 (2)	0.049 (2)	−0.012 (2)	0.008 (2)	−0.0025 (18)
C28	0.065 (3)	0.045 (2)	0.062 (3)	−0.024 (2)	−0.006 (2)	0.000 (2)
C29	0.045 (2)	0.060 (3)	0.056 (3)	−0.0115 (19)	−0.0045 (19)	0.005 (2)
C30	0.045 (2)	0.041 (2)	0.044 (2)	−0.0026 (17)	0.0035 (18)	0.0046 (17)
C31	0.047 (2)	0.0355 (19)	0.040 (2)	0.0033 (16)	0.0017 (17)	−0.0010 (16)
C32	0.074 (3)	0.039 (2)	0.045 (2)	−0.0085 (19)	0.008 (2)	−0.0022 (17)
C33	0.125 (4)	0.036 (2)	0.057 (3)	−0.002 (3)	0.003 (3)	−0.010 (2)
C34	0.099 (4)	0.061 (3)	0.069 (3)	0.028 (3)	0.013 (3)	−0.013 (2)
C35	0.057 (3)	0.077 (3)	0.081 (3)	0.016 (2)	0.016 (2)	−0.018 (3)
C36	0.056 (2)	0.051 (2)	0.065 (3)	0.003 (2)	0.008 (2)	−0.010 (2)
C37	0.045 (2)	0.0334 (19)	0.039 (2)	−0.0050 (16)	0.0016 (17)	−0.0014 (16)
C38	0.052 (2)	0.064 (3)	0.055 (3)	0.013 (2)	0.003 (2)	0.009 (2)
C39	0.070 (3)	0.086 (3)	0.043 (2)	0.011 (3)	0.002 (2)	0.010 (2)
C40	0.069 (3)	0.057 (3)	0.044 (2)	−0.002 (2)	−0.013 (2)	0.0102 (19)
C41	0.064 (3)	0.056 (3)	0.056 (3)	0.014 (2)	−0.009 (2)	0.006 (2)
C42	0.064 (3)	0.053 (2)	0.044 (2)	0.016 (2)	0.002 (2)	0.0012 (19)

### Geometric parameters (Å, º)

**Table:**

Ru1—P1	2.3180 (9)	C17—H17	0.9300
Ru1—P2	2.3493 (9)	C18—H18	0.9300
Ru1—S1	2.4015 (10)	C19—C24	1.386 (4)
Ru1—S2	2.4530 (10)	C19—C20	1.397 (5)
Ru1—S3	2.3981 (9)	C20—C21	1.381 (5)
Ru1—S4	2.4426 (9)	C20—H20	0.9300
S1—C1	1.681 (4)	C21—C22	1.375 (6)
S2—C1	1.672 (4)	C21—H21	0.9300
S3—C4	1.680 (4)	C22—C23	1.368 (6)
S4—C4	1.670 (4)	C22—H22	0.9300
P1—C13	1.830 (3)	C23—C24	1.381 (5)
P1—C19	1.833 (3)	C23—H23	0.9300
P1—C7	1.853 (3)	C24—H24	0.9300
P2—C25	1.841 (3)	C25—C30	1.398 (4)
P2—C31	1.846 (3)	C25—C26	1.402 (4)
P2—C37	1.863 (3)	C26—C27	1.382 (5)
C1—O2	1.342 (4)	C26—H26	0.9300
O2—C3	1.384 (6)	C27—C28	1.379 (5)
C3—H3A	0.9600	C27—H27	0.9300
C3—H3B	0.9600	C28—C29	1.374 (5)
C3—H3C	0.9600	C28—H28	0.9300
C4—O5	1.337 (4)	C29—C30	1.381 (5)
O5—C6	1.430 (5)	C29—H29	0.9300
C6—H6A	0.9600	C30—H30	0.9300
C6—H6B	0.9600	C31—C32	1.382 (5)
C6—H6C	0.9600	C31—C36	1.384 (5)
C7—C8	1.382 (5)	C32—C33	1.396 (5)
C7—C12	1.396 (5)	C32—H32	0.9300
C8—C9	1.385 (5)	C33—C34	1.350 (6)
C8—H8	0.9300	C33—H33	0.9300
C9—C10	1.364 (6)	C34—C35	1.365 (6)
C9—H9	0.9300	C34—H34	0.9300
C10—C11	1.372 (6)	C35—C36	1.383 (5)
C10—H10	0.9300	C35—H35	0.9300
C11—C12	1.381 (5)	C36—H36	0.9300
C11—H11	0.9300	C37—C38	1.379 (5)
C12—H12	0.9300	C37—C42	1.382 (5)
C13—C14	1.381 (5)	C38—C39	1.397 (5)
C13—C18	1.390 (5)	C38—H38	0.9300
C14—C15	1.393 (5)	C39—C40	1.360 (5)
C14—H14	0.9300	C39—H39	0.9300
C15—C16	1.353 (6)	C40—C41	1.363 (5)
C15—H15	0.9300	C40—H40	0.9300
C16—C17	1.376 (6)	C41—C42	1.394 (5)
C16—H16	0.9300	C41—H41	0.9300
C17—C18	1.389 (5)	C42—H42	0.9300
			
P1—Ru1—P2	100.95 (3)	C17—C16—H16	119.7
P1—Ru1—S3	94.66 (3)	C16—C17—C18	120.3 (4)
P2—Ru1—S3	91.54 (3)	C16—C17—H17	119.9
P1—Ru1—S1	94.51 (3)	C18—C17—H17	119.9
P2—Ru1—S1	104.18 (3)	C17—C18—C13	120.0 (4)
S3—Ru1—S1	159.93 (4)	C17—C18—H18	120.0
P1—Ru1—S4	86.97 (3)	C13—C18—H18	120.0
P2—Ru1—S4	162.01 (3)	C24—C19—C20	118.2 (3)
S3—Ru1—S4	71.58 (3)	C24—C19—P1	124.7 (3)
S1—Ru1—S4	91.13 (3)	C20—C19—P1	117.0 (3)
P1—Ru1—S2	163.72 (4)	C21—C20—C19	120.8 (4)
P2—Ru1—S2	90.89 (4)	C21—C20—H20	119.6
S3—Ru1—S2	96.16 (4)	C19—C20—H20	119.6
S1—Ru1—S2	71.57 (4)	C22—C21—C20	119.5 (4)
S4—Ru1—S2	84.96 (4)	C22—C21—H21	120.2
C1—S1—Ru1	86.74 (13)	C20—C21—H21	120.2
C1—S2—Ru1	85.26 (13)	C23—C22—C21	120.7 (4)
C4—S3—Ru1	86.94 (13)	C23—C22—H22	119.7
C4—S4—Ru1	85.69 (12)	C21—C22—H22	119.7
C13—P1—C19	105.04 (15)	C22—C23—C24	120.0 (4)
C13—P1—C7	100.79 (16)	C22—C23—H23	120.0
C19—P1—C7	96.96 (15)	C24—C23—H23	120.0
C13—P1—Ru1	113.81 (11)	C23—C24—C19	120.8 (4)
C19—P1—Ru1	119.25 (11)	C23—C24—H24	119.6
C7—P1—Ru1	118.13 (12)	C19—C24—H24	119.6
C25—P2—C31	103.00 (15)	C30—C25—C26	117.5 (3)
C25—P2—C37	99.82 (14)	C30—C25—P2	122.4 (3)
C31—P2—C37	99.36 (15)	C26—C25—P2	119.9 (3)
C25—P2—Ru1	113.33 (11)	C27—C26—C25	120.7 (3)
C31—P2—Ru1	123.81 (11)	C27—C26—H26	119.7
C37—P2—Ru1	114.11 (11)	C25—C26—H26	119.7
O2—C1—S2	128.0 (3)	C28—C27—C26	120.7 (4)
O2—C1—S1	116.3 (3)	C28—C27—H27	119.7
S2—C1—S1	115.7 (2)	C26—C27—H27	119.7
C1—O2—C3	116.6 (4)	C29—C28—C27	119.6 (4)
O2—C3—H3A	109.5	C29—C28—H28	120.2
O2—C3—H3B	109.5	C27—C28—H28	120.2
H3A—C3—H3B	109.5	C28—C29—C30	120.3 (4)
O2—C3—H3C	109.5	C28—C29—H29	119.8
H3A—C3—H3C	109.5	C30—C29—H29	119.8
H3B—C3—H3C	109.5	C29—C30—C25	121.2 (3)
O5—C4—S4	119.4 (3)	C29—C30—H30	119.4
O5—C4—S3	125.2 (3)	C25—C30—H30	119.4
S4—C4—S3	115.4 (2)	C32—C31—C36	118.0 (3)
C4—O5—C6	117.4 (3)	C32—C31—P2	118.7 (3)
O5—C6—H6A	109.5	C36—C31—P2	123.1 (3)
O5—C6—H6B	109.5	C31—C32—C33	120.0 (4)
H6A—C6—H6B	109.5	C31—C32—H32	120.0
O5—C6—H6C	109.5	C33—C32—H32	120.0
H6A—C6—H6C	109.5	C34—C33—C32	121.2 (4)
H6B—C6—H6C	109.5	C34—C33—H33	119.4
C8—C7—C12	117.4 (3)	C32—C33—H33	119.4
C8—C7—P1	124.4 (3)	C33—C34—C35	119.3 (4)
C12—C7—P1	118.2 (3)	C33—C34—H34	120.3
C7—C8—C9	121.1 (4)	C35—C34—H34	120.3
C7—C8—H8	119.5	C34—C35—C36	120.6 (4)
C9—C8—H8	119.5	C34—C35—H35	119.7
C10—C9—C8	120.6 (4)	C36—C35—H35	119.7
C10—C9—H9	119.7	C35—C36—C31	120.9 (4)
C8—C9—H9	119.7	C35—C36—H36	119.6
C9—C10—C11	119.6 (4)	C31—C36—H36	119.6
C9—C10—H10	120.2	C38—C37—C42	116.8 (3)
C11—C10—H10	120.2	C38—C37—P2	122.0 (3)
C10—C11—C12	120.2 (4)	C42—C37—P2	121.1 (3)
C10—C11—H11	119.9	C37—C38—C39	121.5 (4)
C12—C11—H11	119.9	C37—C38—H38	119.2
C11—C12—C7	121.2 (4)	C39—C38—H38	119.2
C11—C12—H12	119.4	C40—C39—C38	120.5 (4)
C7—C12—H12	119.4	C40—C39—H39	119.7
C14—C13—C18	118.2 (3)	C38—C39—H39	119.7
C14—C13—P1	119.9 (3)	C39—C40—C41	119.0 (4)
C18—C13—P1	121.6 (3)	C39—C40—H40	120.5
C13—C14—C15	121.5 (4)	C41—C40—H40	120.5
C13—C14—H14	119.2	C40—C41—C42	120.7 (4)
C15—C14—H14	119.2	C40—C41—H41	119.7
C16—C15—C14	119.4 (4)	C42—C41—H41	119.7
C16—C15—H15	120.3	C37—C42—C41	121.4 (4)
C14—C15—H15	120.3	C37—C42—H42	119.3
C15—C16—C17	120.6 (4)	C41—C42—H42	119.3
C15—C16—H16	119.7		
			
Ru1—S2—C1—O2	−171.8 (4)	C19—C20—C21—C22	0.0 (6)
Ru1—S2—C1—S1	7.8 (2)	C20—C21—C22—C23	−1.8 (7)
Ru1—S1—C1—O2	171.7 (3)	C21—C22—C23—C24	1.7 (7)
Ru1—S1—C1—S2	−7.9 (2)	C22—C23—C24—C19	0.1 (6)
S2—C1—O2—C3	6.5 (6)	C20—C19—C24—C23	−1.8 (5)
S1—C1—O2—C3	−173.1 (4)	P1—C19—C24—C23	−178.6 (3)
Ru1—S4—C4—O5	−173.8 (3)	C31—P2—C25—C30	40.4 (3)
Ru1—S4—C4—S3	5.8 (2)	C37—P2—C25—C30	142.5 (3)
Ru1—S3—C4—O5	173.6 (3)	Ru1—P2—C25—C30	−95.7 (3)
Ru1—S3—C4—S4	−5.9 (2)	C31—P2—C25—C26	−145.7 (3)
S4—C4—O5—C6	−178.2 (3)	C37—P2—C25—C26	−43.6 (3)
S3—C4—O5—C6	2.2 (5)	Ru1—P2—C25—C26	78.1 (3)
C13—P1—C7—C8	−118.0 (3)	C30—C25—C26—C27	0.6 (5)
C19—P1—C7—C8	135.2 (3)	P2—C25—C26—C27	−173.6 (3)
Ru1—P1—C7—C8	6.6 (4)	C25—C26—C27—C28	−1.0 (5)
C13—P1—C7—C12	60.5 (3)	C26—C27—C28—C29	0.4 (6)
C19—P1—C7—C12	−46.3 (3)	C27—C28—C29—C30	0.7 (6)
Ru1—P1—C7—C12	−174.9 (2)	C28—C29—C30—C25	−1.1 (6)
C12—C7—C8—C9	0.3 (6)	C26—C25—C30—C29	0.5 (5)
P1—C7—C8—C9	178.8 (3)	P2—C25—C30—C29	174.4 (3)
C7—C8—C9—C10	−0.2 (7)	C25—P2—C31—C32	−158.7 (3)
C8—C9—C10—C11	−0.3 (7)	C37—P2—C31—C32	98.9 (3)
C9—C10—C11—C12	0.5 (6)	Ru1—P2—C31—C32	−28.6 (3)
C10—C11—C12—C7	−0.4 (6)	C25—P2—C31—C36	27.6 (3)
C8—C7—C12—C11	0.0 (6)	C37—P2—C31—C36	−74.8 (3)
P1—C7—C12—C11	−178.6 (3)	Ru1—P2—C31—C36	157.7 (3)
C19—P1—C13—C14	−59.5 (3)	C36—C31—C32—C33	−2.1 (6)
C7—P1—C13—C14	−159.8 (3)	P2—C31—C32—C33	−176.2 (3)
Ru1—P1—C13—C14	72.7 (3)	C31—C32—C33—C34	2.3 (6)
C19—P1—C13—C18	127.7 (3)	C32—C33—C34—C35	−0.4 (7)
C7—P1—C13—C18	27.4 (3)	C33—C34—C35—C36	−1.7 (7)
Ru1—P1—C13—C18	−100.1 (3)	C34—C35—C36—C31	1.8 (7)
C18—C13—C14—C15	−1.2 (5)	C32—C31—C36—C35	0.1 (6)
P1—C13—C14—C15	−174.2 (3)	P2—C31—C36—C35	173.8 (3)
C13—C14—C15—C16	0.9 (6)	C25—P2—C37—C38	−81.6 (3)
C14—C15—C16—C17	−0.3 (7)	C31—P2—C37—C38	23.5 (3)
C15—C16—C17—C18	0.1 (7)	Ru1—P2—C37—C38	157.2 (3)
C16—C17—C18—C13	−0.4 (6)	C25—P2—C37—C42	95.0 (3)
C14—C13—C18—C17	0.9 (6)	C31—P2—C37—C42	−159.9 (3)
P1—C13—C18—C17	173.9 (3)	Ru1—P2—C37—C42	−26.2 (3)
C13—P1—C19—C24	−9.2 (3)	C42—C37—C38—C39	0.1 (6)
C7—P1—C19—C24	94.0 (3)	P2—C37—C38—C39	176.8 (3)
Ru1—P1—C19—C24	−138.3 (3)	C37—C38—C39—C40	−0.3 (6)
C13—P1—C19—C20	173.9 (3)	C38—C39—C40—C41	0.8 (6)
C7—P1—C19—C20	−82.9 (3)	C39—C40—C41—C42	−1.1 (6)
Ru1—P1—C19—C20	44.9 (3)	C38—C37—C42—C41	−0.3 (5)
C24—C19—C20—C21	1.7 (5)	P2—C37—C42—C41	−177.1 (3)
P1—C19—C20—C21	178.8 (3)	C40—C41—C42—C37	0.9 (6)

### Hydrogen-bond geometry (Å, º)

Cg1 is the centroid of the C25–C30 ring.

**Table:**

*D*—H···*A*	*D*—H	H···*A*	*D*···*A*	*D*—H···*A*
C11—H11···O5^i^	0.93	2.51	3.387 (5)	157
C40—H40···*Cg*^ii^	0.93	2.85	3.521 (4)	130

Symmetry codes: (i) *x*−1/2, −*y*+1/2, −*z*; (ii) *x*+1/2, *y*, −*z*+1/2.

## References

[bb1] Bag, N., Lahiri, G. K. & Chakravorty, A. (1990). *J. Chem. Soc. Dalton Trans.* pp. 1557–1561.

[bb2] Brandenburg, K. (2006). *DIAMOND* Crystal Impact GbR, Bonn, Germany.

[bb3] Bruker (2007). *APEX2*, *SAINT* and *SADABS* Bruker AXS Inc., Madison, Wisconsin, USA.

[bb4] Farrugia, L. J. (2012). *J. Appl. Cryst.* **45**, 849–854.

[bb5] Liu, X., Zhang, Q.-F. & Leung, W.-H. (2005). *J. Coord. Chem.* **58**, 1299–1305.

[bb6] Lu, X. L., Ng, S. Y., Vittal, J. J., Tan, G. K., Goh, Y. L. & Hor, T. S. A. (2003). *J. Organomet. Chem.* **688**, 100–111.

[bb7] Noda, K., Ohuchi, Y., Hashimoto, A., Fujiki, M., Itoh, S., Iwatsuki, S., Noda, T., Suzuki, T., Kashiwabara, K. & Tagagi, H. D. (2006). *Inorg. Chem.* **45**, 1349–1355. 10.1021/ic051487l 16441147

[bb8] Sheldrick, G. M. (2008). *Acta Cryst.* A**64**, 112–122. 10.1107/S0108767307043930 18156677

[bb9] Spek, A. L. (2009). *Acta Cryst.* D**65**, 148–155. 10.1107/S090744490804362X PMC2631630 19171970

[bb10] Wang, X.-Y., Li, Y., Ma, Q. & Zhang, Q.-F. (2010). *Organometallics*, **29**, 2752–2760.

[bb11] Wu, F.-H., Duan, T., Lu, L., Zhang, Q.-F. & Leung, W.-H. (2009). *J. Organomet. Chem.* **694**, 3844–3851.

